# Observation Series-Based Skymask Establishment and NLOS Exclusion for Smartphone Positioning

**DOI:** 10.3390/s26072140

**Published:** 2026-03-30

**Authors:** Chao Liu, Ke Wu

**Affiliations:** School of Spatial Information and Geomatics Engineering, Anhui University of Science and Technology, Huainan 232001, China; 2023201554@aust.edu.cn

**Keywords:** GNSS, NLOS detection, smartphones, skymask, sky visibility

## Abstract

Detecting non-line-of-sight (NLOS) signals is essential for improving the accuracy and reliability of smartphone Global Navigation Satellite System (GNSS) positioning in dense urban areas. This paper presents a practical method for NLOS detection based on skymasks derived from smartphone observations. The observable rates of satellite observation series are first computed using precise ephemeris, and the observations are then classified into blocked and unblocked groups. A smoothing spline is then applied to fit the building boundary from the categorized series. Based on the fitted boundary, a skymask is constructed and used for NLOS detection. Datasets collected at three locations using three different smartphones are used for validation. The results show that both the number and proportion of NLOS signals decrease significantly after applying the proposed method. As the degree of obscuration increases, the detection accuracy remains stable across different smartphones. In some cases, single-point positioning accuracy is improved after excluding NLOS signals. In addition, the derived skymask can be used to estimate sky visibility and support the selection of positioning strategies. Overall, the proposed method can be combined with the consistency checking method for NLOS detection, as it does not require additional information.

## 1. Introduction

Location-based services (LBSs) are widely used in applications such as personal navigation, geolocation apps, and ride-sharing [1]. For LBS, accurate positioning is a crucial task, as it forms the foundation of many LBS applications [2]. Since satellite navigation chipsets are embedded in smartphones and can conveniently provide absolute positioning information, they play a vital role in LBS applications. In 2016, Google announced that raw GNSS measurements would be accessible on Android devices [3]. This enables the development of precise positioning algorithms to improve the robustness and availability of the LBSs on Android smartphones.

However, providing satisfactory LBS performance with smartphones in urban areas remains challenging because GNSS signals can be blocked or reflected by tall buildings and other obstacles in these areas. These effects are commonly referred to as multipath and non-line-of-sight (NLOS) propagation, which degrades positioning accuracy and limits positioning performance. Two types of signal propagation can be distinguished according to the reception conditions [4,5,6,7]. If the smartphone receives both direct and reflected signals simultaneously, the phenomenon is referred to as line-of-sight (LOS) multipath, and the resulting ranging error is called LOS multipath error. If the smartphone only receives indirect signals reflected or distracted by obstacles, these signals are referred to as NLOS signals. Due to the signal processing capability of modern GNSS receivers, the error caused by LOS multipath in pseudorange measurements is usually limited to a few meters. However, the error caused by NLOS cannot be characterized by a fixed magnitude since it depends on the observed environment. Positioning errors may exceed 50 m when NLOS signals are used [8]. Detecting and excluding NLOS signals is an effective way to improve the smartphone positioning performance in urban areas. Before performing NLOS signal processing, such as exclusion or correction, it is essential to detect NLOS observations in each epoch. A large body of research has been devoted to detecting NLOS signals in urban environments. Existing approaches mainly differ in the type of information they use and the level of environmental knowledge required.

One straightforward strategy relies on signal-quality indicators such as the signal-to-noise ratio (SNR) or carrier-to-noise density ratio (C/N0) to identify potentially blocked signals [9,10]. Because of their simplicity, these methods are easy to implement and have been widely used in low-cost GNSS applications. However, signal strength is influenced not only by obstruction but also by device hardware, antenna characteristics, and surrounding environments, which limits the robustness of fixed-threshold strategies across different smartphones and urban scenarios [5,11].

Another widely used approach relies on the internal consistency of GNSS observations. By examining whether pseudorange measurements are compatible with the positioning geometry, methods based on consistency checking, such as RAIM or residual-based detection, can effectively identify abnormal observations when the number of contaminated signals is limited [12,13,14,15]. Nevertheless, in dense urban environments where multiple NLOS signals may occur simultaneously, the consistency relationships among observations can be severely distorted, which reduces the effectiveness of such approaches [16,17].

To improve environmental awareness, several studies incorporate explicit scene information into the detection process. A representative example is the use of 3D map-aided (3DMA) techniques, which infer satellite visibility from building models or urban maps and then classify observations accordingly [18,19,20,21,22]. Related work has also introduced complementary sensing devices, such as fisheye cameras or LiDAR, to directly extract skyline or obstruction information [23,24,25,26]. Although these approaches can provide strong geometric constraints, their applicability depends on the availability, completeness, and positional accuracy of external data sources, which may be limited in many real-world smartphone positioning scenarios [1,27,28].

In recent years, machine learning-based approaches have been increasingly explored for LOS/NLOS classification. By learning signal patterns from observation features, algorithms such as support vector machines (SVM), decision trees, and convolutional neural networks have shown promising performance in various studies [4,29,30,31]. However, these methods typically require sufficiently representative labeled datasets, and their generalization capability may be affected by differences in smartphone hardware, signal quality, and environmental characteristics [6,32,33,34,35]. Nevertheless, recent studies have shown that lightweight machine learning models can also provide efficient LOS/NLOS classification with relatively low computational cost. Integrating such lightweight learning-based approaches with observation-series-based methods may further improve NLOS detection performance in complex urban environments and could be an interesting direction for future research.

Considering the limitations of the above approaches, there remains a need for a practical NLOS detection strategy that does not rely on additional environmental information. To address this issue, this paper proposes a skymask establishment method for smartphone positioning that does not require extra equipment or external environmental information. In this method, the satellites that could theoretically be observed are first determined using precise ephemeris, and the observable rate of each satellite observation series is then calculated. Based on the observable rate, the observation series are categorized into blocked and unblocked series. The building boundary is then fitted using the categorized observations and a smoothing spline model. Finally, NLOS signals are detected using the skymask extracted from the fitted building boundary. The effectiveness of the proposed skymask establishment and NLOS detection method is verified using real smartphone datasets. The remainder of this paper is organized as follows. Section 2 presents an overview of the proposed method, and then the performance of the method is discussed in Section 3. Section 4 notes conclusions and future work.

## 2. Building Boundary Establishment

In this paper, a building boundary is established using time-series observations from Android smartphones, and then the boundary is applied to detect the NLOS observations. The three steps in the establishment include observation series classification, boundary fitting, and boundary rectification.

### 2.1. Observation Series Classification

Generally, all satellite signals can be classified into two types: blocked signals and unblocked signals. For the unblocked satellite, the signal is directly received by the smartphone, which means that the elevation of the satellite exceeds the highest elevation of the surrounding building edges over the same azimuth. Assuming that all the unblocked signals can be acquired by the smartphone, and the sky outline can be estimated from unblocked signals, which provide elevation information without blockage. In contrast, the signal blocked by the building means that the elevation of these satellites is smaller than the building edges, and the building outline can be estimated from the signal. Combined with the sky outline and building outline, an approximate building boundary can be obtained.

Therefore, the first step is to classify the satellite signals into the above two categories. With the open API and network capabilities, the approximate position can be obtained, as well as a precise ephemeris, from a smartphone. Furthermore, the azimuth and elevation of all satellites can be calculated. Even if a satellite is not tracked by the smartphone, its theoretical sky position and visibility can still be determined from the satellite ephemeris and the approximate receiver position.

**Figure 1:**
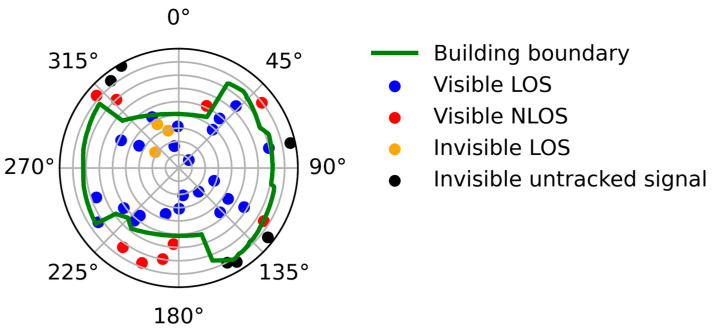
Position of different satellites. The green line is the building boundary, blue points are visible satellite signals, red points are visible NLOS signals, orange points are invisible LOS signals, and black points are untracked signals that are outside the boundaries.

For instance, all satellites in an epoch are shown in Figure 1, and the green line is the building boundary, which is extracted from the picture of a fisheye camera. Four types of signals are shown in the figure: visible LOS, visible NLOS, invisible LOS, and invisible signals blocked by buildings. The figure shows that some LOS signals with high elevation cannot be received by the smartphone; therefore, it is unreliable to treat the untracked satellites as NLOS signals as described in [32]. Searching for the blocked and unblocked signals with observations in an epoch is difficult. In this paper, we apply the time-series observation to achieve the data separation, taking into consideration that the observable rate of the LOS signal series is high while the rate of NLOS is low.

**Figure 2:**
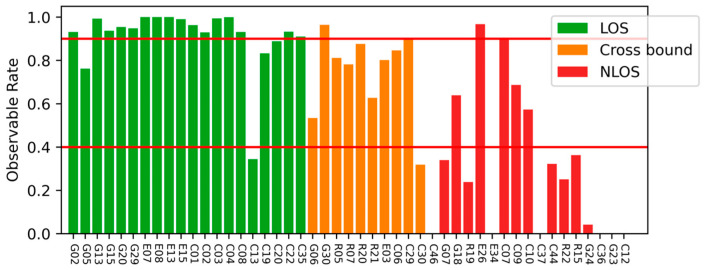
Observable rate of different satellites. Green bars are LOS signal series over the period, orange bars are signal series that cross the building boundaries, and red bars are NLOS signal series over the period. Red lines are thresholds.

Figure 2 shows the observable rate of satellite observation series; the green bar is the series that is unblocked over the period, the orange bar is the series whose trajectory crosses the building boundaries, and the red bar is the observation series that is blocked over the period. The red lines represent the thresholds of 0.9 and 0.4. We treat observation series with values greater than 0.9 as unblocked series, and observation series with observable rates lower than 0.4 as blocked series. It can be observed that most blocked and unblocked series can be separated by this threshold. We did not process the series that could not be judged to avoid the influence of misjudgment.

In practice, satellites with observable rates close to 1.0 generally correspond to clear line-of-sight visibility, while heavily obstructed satellites often exhibit significantly lower observable rates due to frequent signal interruptions. Based on empirical observations of the datasets used in this study, thresholds of 0.9 and 0.4 were adopted to identify unblocked and blocked observation series, respectively. Observation series with observable rates between these values were excluded from boundary fitting to avoid ambiguous cases and improve the robustness of the building boundary estimation.

**Figure 3:**
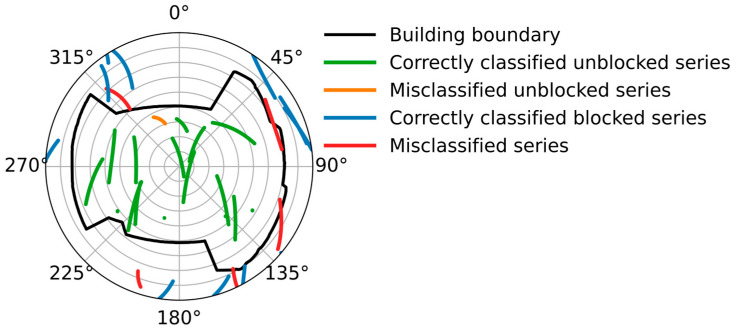
Trajectory of satellites. Green and blue lines indicate correctly classified unblocked and blocked signal series, respectively, while red lines indicate misclassified series.

Figure 3 shows the trajectory of different separated satellites, in which green lines are unblocked signals, blue lines are blocked signals, and red lines are the signals that are misjudged. The figure depicts that there is sufficient data in different azimuths, which are correctly classified, and are beneficial for boundary fitting. In addition, only six series are misclassified in the figure. Note that the trajectory of these data is near the building boundaries, and they do not cause a serious influence on the boundary fitting.

It should be noted that the proposed skymask is derived from satellite observation statistics rather than explicit building models. Therefore, the fitted boundary represents an effective obstruction boundary reflected in satellite visibility data and may include the influence of various surrounding obstacles, such as buildings and dense vegetation. If certain obstacles consistently and significantly block satellite signals along specific azimuth directions, their effects are more likely to be reflected in reduced observable rates and thus incorporated into the boundary estimation.

However, for transient or semi-transparent obstacles, such as sparse foliage, moving branches, or seasonal vegetation changes, the impact on satellite visibility may be weaker or less stable over time. In such cases, the observable rate may fluctuate, and the corresponding effects may not be consistently captured in the fitted boundary. This limitation should be considered when applying the proposed method in environments with dynamic or weak obstructions.

### 2.2. Boundary Fitting

The reliability of the fitted building boundary depends on the temporal coverage of the satellite observation series. In practice, a sufficiently long observation period is required so that satellite trajectories can provide adequate azimuth coverage for both blocked and unblocked observation series. If the observation duration is too short, large azimuth gaps may occur, which can reduce the robustness of the fitted boundary. In this study, more than 2 h of static observations at a sampling rate of 1 Hz were collected at each test location to ensure stable satellite trajectories and sufficient data for the boundary fitting process.

Blocked observation series and unblocked observation series can be obtained based on the classification method in Section 2.1. The next process is to combine the two series for boundary fitting. Note that the azimuth interval is set as 1° in the data combination.

For blocked signals, the elevation angle of the satellites is smaller than the building edge over the azimuth, and the signal with the highest elevation is closest to the building edge. Analogously, the signals from unblocked satellites have elevations higher than the building edge, and we should choose the signal with the smallest elevation over the azimuth. For the azimuth interval that contains a blocked signal and an unblocked signal simultaneously, we fuse the two signals with a weighted strategy as follows:

(1) $$ele_{i}=\sqrt{\frac{ele_{blocked,\;i}}{90}}\times ele_{blocked}+\left(1-\sqrt{\frac{ele_{unblocked,\;i}}{90}}\right)\times ele_{unblocked}$$

where the i is the ith azimuth interval; $ele_{blocked,i}$ and $ele_{unblocked}$ are elevation angles of blocked and unblocked signals in the ith azimuth; and the fused elevation $ele_{i}$ is used to represent the effective boundary observation within the i-th azimuth interval by combining blocked and unblocked signal information. It should be noted that a blocked signal’s elevation might exceed that of an unblocked signal in certain cases. In such instances, the blocked satellite is prioritized as the fitting element to ensure a stricter building boundary and minimize the impact of potential misclassification. The coefficients in Equation (1) are not constrained to sum to one because the equation is intended as a heuristic fusion rule rather than a strict weighted average. This design biases the fused elevation toward a more conservative obstruction boundary and helps avoid underestimation when blocked and unblocked observations are inconsistent. Similar to the method described in [33], a smoothing spline is applied to fit the boundary, and the function of the spline is as follows:

(2) $$f\left(x\right)=\min\left(p\sum_{i}w_{i}{\left(ele_{i}-f\left({azi}_{i}\right)\right)}^{2}+\left(1-p\right)\int_{0{}^{\circ}}^{360{}^{\circ}}{\left(\frac{d^{2}f\left(x\right)}{dx^{2}}\right)}^{2}dx\right)$$

where f(x) is the fit spline, $azi_{i}$ is the azimuth in the ith interval and $ele_{i}$ is its elevation angle, and p denotes a smoothing parameter that is tuned heuristically. $w_{i}$ is the weighting, given the satellite i, and it is calculated as below:

(3) $$w_{i}=\sqrt{1-\frac{ele_{i}}{90}}$$

Equations (2) and (3) define the smoothing-spline fitting process and the corresponding weighting strategy used to estimate a continuous building boundary from the selected observations. The smoothing parameter p controls the trade-off between fitting accuracy and smoothness of the estimated boundary. A moderate value was selected empirically to avoid overfitting to irregular elevation variations while preserving the overall skyline structure. In this paper, the boundary is fit by the observation series, and it is more intensive compared to the method described in [33]. However, large azimuth gaps between adjacent satellites may occur, which can negatively affect the boundary estimation. The threshold of the azimuth gap is set to 15°. When the gap is over the threshold, we use the hypothesized virtual satellites and set their elevation angles by linear interpolation.

**Figure 4:**
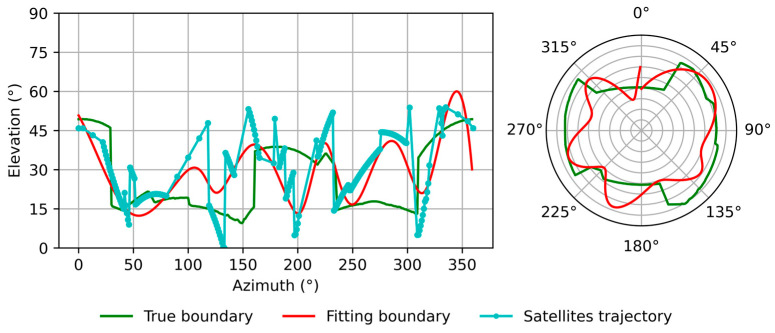
Estimated building boundaries. The green line is the real boundary, the cyan line is the position of satellites, and the red line is the fit boundary.

Figure 4 shows the fitting result based on the method mentioned above. The green line is the real boundary, the cyan line is the selected satellites, and the red line is the fitting boundary. It can be observed that the fitted boundary generally follows the real boundary. The difference between the fitted boundary and the true boundary mainly results from the discrete distribution of satellite observations and the limited number of available satellites in some azimuth intervals. Despite these deviations, the fitted boundary still captures the dominant obstruction pattern required for NLOS detection.

### 2.3. Boundary Rectification

There could be an inconsistency between the fit boundary and classified satellites caused by the features of the smoothing spline. For instance, the elevation of the unblocked satellite can be smaller than the boundary over the same azimuth, while the elevation of a blocked satellite can be greater than the boundary. It will degrade the reliability of the fit boundary and cause a negative effect on NLOS detection. Therefore, rectifying the fit boundary is necessary.

There are two steps in boundary rectification, including resampling and optimization. The resampling step checks all selected satellites used for fitting and adjusts the inconsistent observations. The optimization step is to optimize the fit boundary based on adjusted satellites. The equation for the inconsistent satellite adjustment is as follows:

(4) $$ele_{j,\;adjust}=\left\{\begin{array}{c} \begin{array}{cc} f\left(azi_{j}\right)-ele_{bias} & ele_{j}<f\left(azi_{j}\right),\;s_{j}\;is\;unblocked \end{array}\\ \begin{array}{cc} f\left(azi_{j}\right)+ele_{bias} & ele_{j}>f\left(azi_{j}\right),\;s_{j}\;is\;blocked \end{array} \end{array}\right.$$

where $ele_{j,adjust}$ is the adjusted elevation of the virtual satellite obtained from the original inconsistent satellite $s_{j}$ with the elevation $ele_{j}$, and $f(azi_{j})$ is the elevation of the fit boundary over the azimuth of $s_{j}$. $ele_{bias}$ is the adjusted bias and is set as 5° in this paper. Equation (4) is used to adjust observations that are inconsistent with the fitted boundary, thereby improving the reliability of the final skymask. In addition, inconsistent satellites with elevations smaller than 20° would be neglected in adjustment, since they would not affect positioning significantly after excluding these satellites.

**Figure 5:**
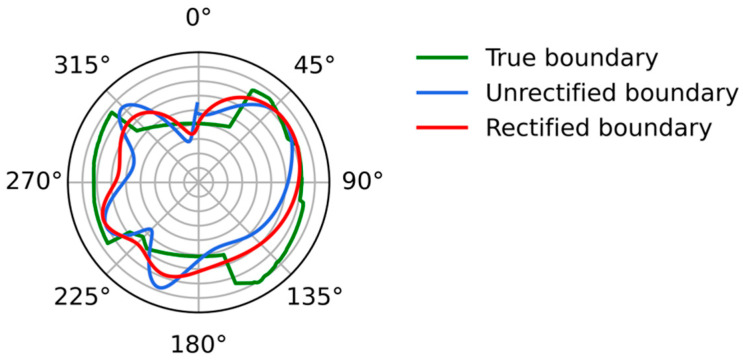
Comparison of different boundaries. The green line is the real boundary, the blue line is the unrectified boundary, and the red line is the rectified boundary.

Figure 5 shows the two fit boundaries. The green line is the real boundary, the blue line is the boundary without rectification, and the red line is the rectified boundary. It can be observed from the figure that the rectified boundary is more reliable.

## 3. Results

In this section, we analyze the performance of the method, including the NLOS signals detection, sky visibility estimation, and single-point positioning after NLOS elimination by the method on three smartphones.

### 3.1. Experiment Setup

**Figure 6:**
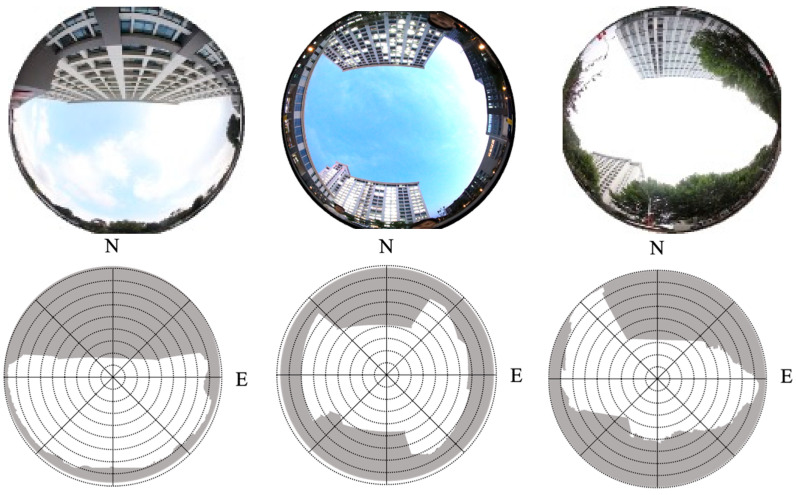
Image from a fisheye camera in three locations. P1 (**left**); P2 (**middle**); P3 (**right**).

To verify the NLOS detection effectiveness of the proposed method, we performed a static experiment at three locations in Southeast University, Jiangsu, China. The pictures captured by a fisheye camera at different points are shown in Figure 6 (top). It should be noted that the fisheye camera was used only to obtain the reference building boundary for evaluation purposes. It was not used as an input to the proposed skymask establishment or NLOS detection method, which relies solely on GNSS observations and satellite ephemeris information. For the first point, smartphones are put close to the building, and half the sky is blocked. The second location is surrounded by buildings, and two of them are higher than 50 m. The third location is on a road that is blocked by two tall buildings and some trees. The locations are named P1, P2, and P3 for a convenient expression.

**Figure 7:**
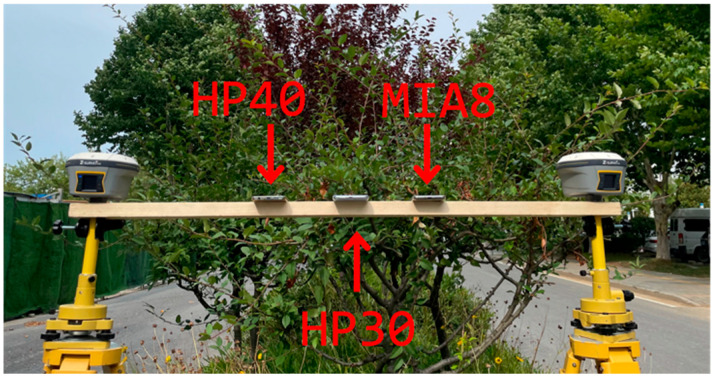
Relative position of geodetic receivers and three smartphones.

At each location, three smartphones were used to collect GNSS observations for more than 2 h with a sampling interval of 1 s, and the observations were collected by Geo++ (v2.1.8). Information about the smartphones used is shown in Table 1. The smartphones were placed between two Huace i50 geodetic receivers (Shanghai Huace Navigation Technology Ltd., Shanghai, China), which can collect observations for reference position calculation, as Figure 7.

### 3.2. Number of NLOS Signals

If NLOS signals are rare in a given epoch, positioning will not be seriously degraded because the consistency-check method can still detect and exclude a small number of NLOS satellites. However, the effect of this method is not ideal when there are several NLOS signals. In this case, we discuss the NLOS exclusion performance of the proposed method by analyzing the number of NLOS signals in an epoch.

**Figure 8:**
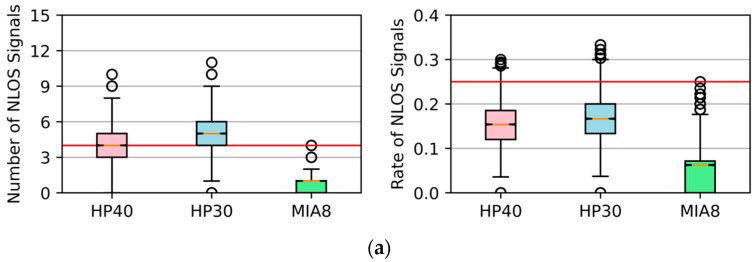

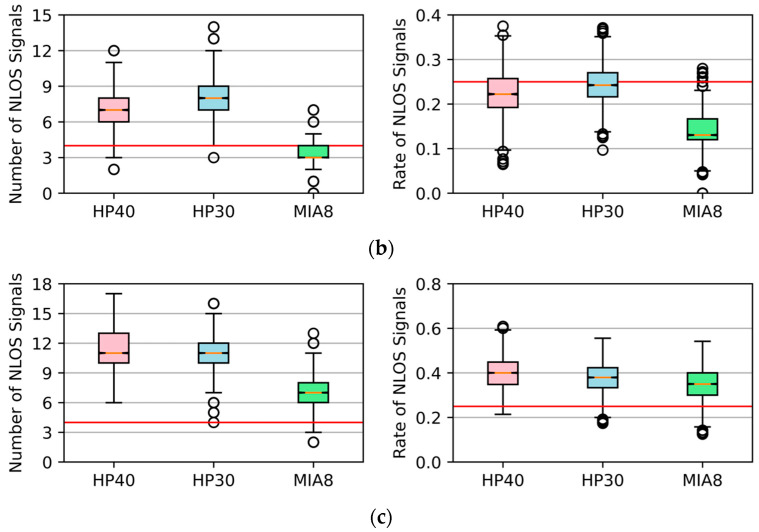
The number and rate of NLOS signals in an epoch in different locations. (**a**) P1, open-sky or weak-obstruction location; (**b**) P2, location close to tall buildings; (**c**) P3, road between buildings. Pink boxes for HP40, blue boxes for HP30, and green boxes for MIA8.

Figure 8 shows the number and the rate of NLOS signals on one observation epoch in three different locations using a boxplot. In the boxplots, the central horizontal line represents the median value, the box indicates the interquartile range, the whiskers denote the non-outlier range, and the circles represent outliers. The red horizontal reference lines are added to facilitate visual comparison of the distributions. Compared to Huawei smartphones, the Xiaomi Mi8 received fewer NLOS signals in all locations. In P1, there was typically one NLOS signal per epoch, and its proportion was lower than 10%. If the smartphone was surrounded by tall buildings, as in P2, the number of NLOS signals increased to about three, and the rate exceeded 10%. When the smartphone was positioned on a road between buildings, as in P3, there were always more than six NLOS signals in an epoch, the rate exceeded 25%, and the consistency check could not work theoretically. For Huawei smartphones, the number of NLOS signals in an epoch exceeded three in all three collecting locations. In P2 and P3, more than six NLOS signals could be received by the HP30 and HP40 every second, and the rate exceeded 20% in most cases. Overall, the three smartphones were affected by NLOS signals significantly in the test locations close to tall buildings.

**Figure 9:**
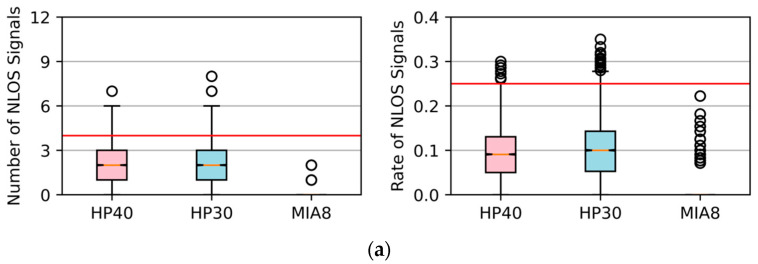

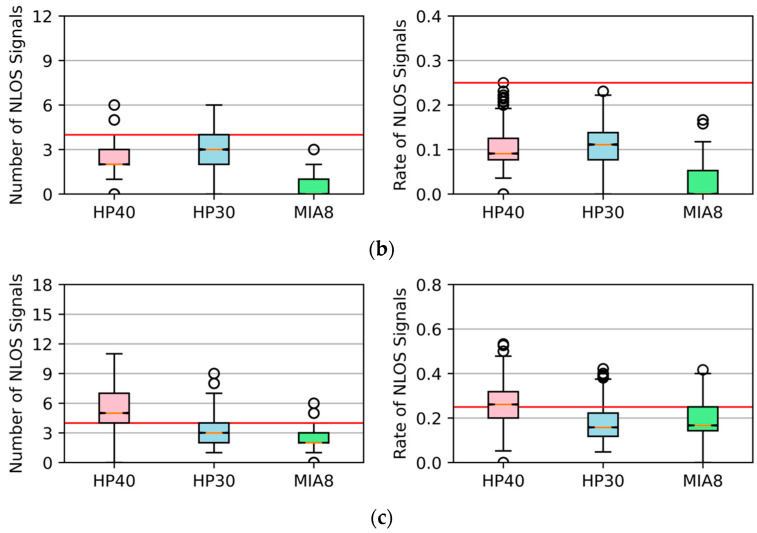
The number and rate of NLOS signals in an epoch after NLOS excluding by the proposed method. (**a**) P1, open-sky or weak-obstruction location; (**b**) P2, location close to tall buildings; (**c**) P3, road between buildings.

Using the proposed method, we established skymasks epoch by epoch and then applied them to detect and exclude NLOS signals. The number of remaining NLOS signals and the rate every second are depicted in Figure 9.

Compared with Figure 8, the number of NLOS signals for all smartphones decreased significantly. For MIA8, there are fewer than three NLOS signals in most epochs in all test locations. For HP30, the number of NLOS signals was smaller than four, and the rate was close to 10%. A similar result could be obtained from observations of HP40 in P1 and P2. However, the detection in P3 was not ideal, and there were still five NLOSs in the observation. This could be caused by the unsatisfactory skymask fitting. Overall, the proposed skymask could detect most NLOS signals, and we could combine this method with the consistency check to exclude the remaining small amount of NLOS signals.

### 3.3. Accuracy of Detection

Four types of outcomes may occur after applying an NLOS detection method to a satellite, including (1) true positive (TP) that detects the LOS signal correctly; (2) true negative (TN) that detects the NLOS signal correctly; (3) false positive (FP), in which an NLOS signal is incorrectly classified as LOS; (4) false negative (FN), in which an LOS signal is incorrectly classified as NLOS. The detection accuracy can then be calculated as follows:

(5) $$Accuracy=\frac{TP+TN}{TP+TN+FP+FN}$$

In addition, the influence on the positioning of FP and FN is different. For FN, we could exclude LOS signals, and it will degrade positioning by decreasing the DOP value. For FP, the NLOS signal could be reserved and applied in positioning. Comparably, the impact of FP could be more serious.

**Figure 10:**
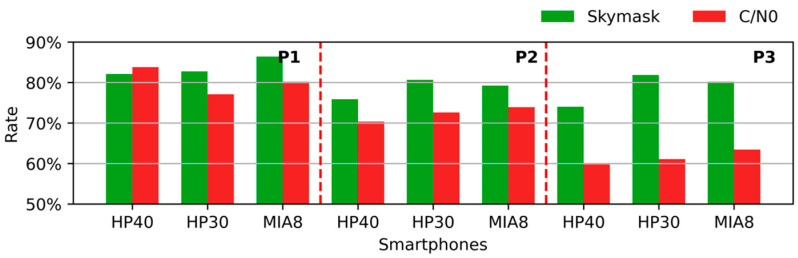
Detection accuracy in different locations. Green bars are the results of the proposed method, and red bars are the detection accuracies of the C/N0 threshold method.The red dotted lines separate the results for the three test locations, namely P1, P2, and P3.

We used the NLOS detection result based on the C/N0 threshold as a reference, since this method is also free of extra information. Additionally, we set the C/N0 threshold as 30 dB-Hz. Figure 10 shows the detection accuracy of three smartphones in three test locations. The green bar is the result of the proposed method, which is named skymask in the legend, and the red bar is the result of the C/N0 threshold method, which is named C/N0 in the legend.

In P1, where the smartphones blocked half the sky, the detection accuracy of the proposed method exceeded 80% of all smartphones, and the accuracy of the control method exceeded 75%. Specifically, the detection accuracy of the skymask was higher than that of the C/N0 threshold method on HP30 and MIA8. It was worse on HP40, but the difference was smaller than 1.5%. When smartphones were surrounded by buildings, as in P2 and P3, the detection accuracy of the proposed method was stable, and the value exceeded 75% of all smartphones. However, the effectiveness of the C/N0 method was unsatisfactory. It should be noted that with the increased degree of obscuration, the detection accuracy of the C/N0 method decreased significantly, while it was stable with the proposed method.

**Figure 11:**
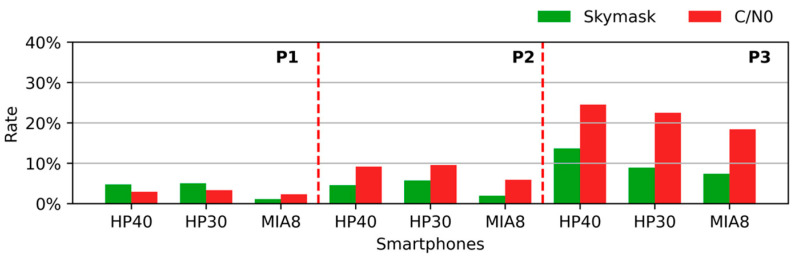
FP rates of different methods at three test locations. The green bars represent the results of the proposed skymask method, and the red bars represent the results of the C/N0-threshold method. The red dotted lines separate the results for the three test locations, namely P1, P2, and P3.

It has been discussed that a lower FP rate is preferable, and the value of FP in the three locations is shown in Figure 11. It can be observed that the FP rate increased with the degree of obscuration. For the proposed method, the rate of FP was lower than 10% in most cases. However, this value could reach more than 20% by the C/N0 threshold method. In this case, more NLOS signals can be excluded by the proposed method. Overall, if we could not provide extra information for NLOS detection, better detection effectiveness could be obtained by the proposed method in this paper compared with the C/N0 method.

### 3.4. Sky Visibility Estimation

**Figure 12:**
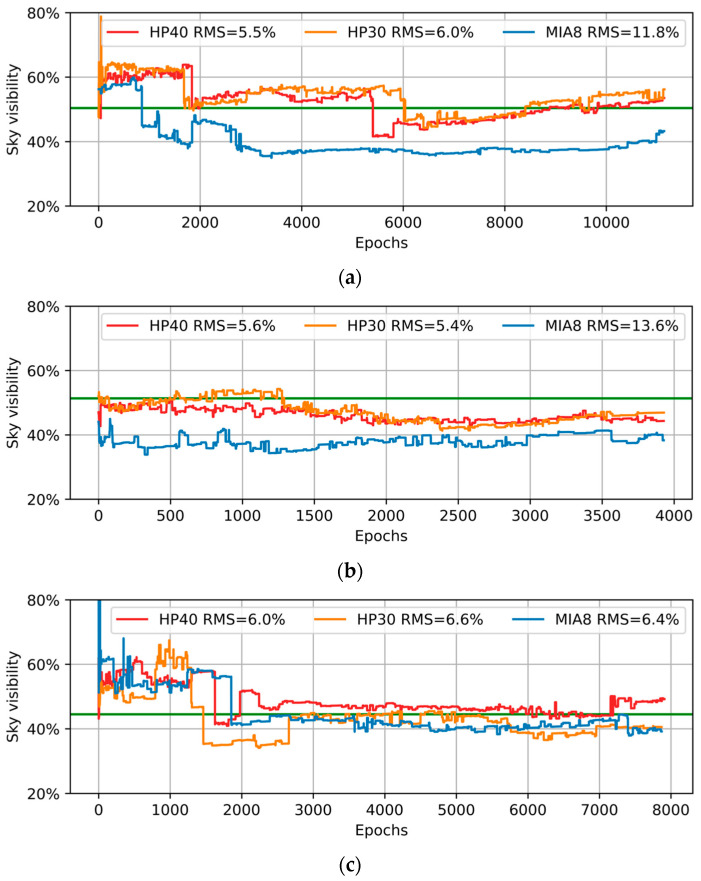
Results of sky visibility estimation in different locations. (**a**) P1, open-sky or weak-obstruction location; (**b**) P2, location close to tall buildings; (**c**) P3, road between buildings. In each subfigure, the green line denotes the true sky visibility, while the red, orange, and blue lines denote the estimated results of HP40, HP30, and MIA8, respectively.

Sky visibility can be treated as an indicator of the degree of obscuration, and we can select different methods to improve positioning accuracy based on this indicator [33]. For instance, the WLS method can be applied in an open-sky area with large sky visibility. And if we stay in an urban area where the sky visibility is low, the ray-tracing method could be better. Since the skymask can be derived by this approach, we can also estimate the sky visibility using this skymask. The visibility can be calculated as below:

(6) $$sky\_visi=\sum_{azi=1}^{360}\frac{\left(90\;-\;ele_{i}^{2}\right)}{360*90^{2}}$$

where azi is azimuth and ele is the elevation in degrees. The sky visibility estimation results in three locations are shown in Figure 12.

The figure shows that the estimated effectiveness of Huawei smartphones is stable at around 6%. For MIA8, satisfactory sky visibility can be obtained in P3, and the RMSE is 6.4%. However, the RMS of sky visibility exceeds 10% in P1 and P2, and, intuitively, the estimated result is smaller than the true value. This may be attributed to the insufficient number of observation satellites. In comparison, HP40 and HP30 can receive sufficient satellite signals, and the estimated skymask is more accurate.

### 3.5. Positioning Results and Discussion

In this section, we evaluate the positioning performance with NLOS signals excluded by the proposed method. Since only pseudorange measurements can be received by some smartphones, the single point positioning (SPP) method is applied in this part. In positioning, the satellite position and clock are calculated by broadcast ephemeris, ionospheric delays are estimated by the Klobuchar model, and tropospheric delays are estimated by the Saastamoinen model. We use the stochastic model based on C/N0 to estimate the observation noise. The positioning results of three smartphones in different locations are shown in Table 2. Here, CC denotes the result after applying the consistency-check method, and Skymask is the result based on the proposed method. It should be noted that the consistency method is also applied in this case for removing remaining NLOS signals. Delete All denotes the case where all NLOS signals are removed using the reference skymask extracted from fisheye images. Imp is the improvement between “Skymask” and “CC”. Compared with the CC-only solution, the proposed skymask-based method provides additional improvement by excluding a larger proportion of NLOS-contaminated observations before positioning.

It can be observed from Table 2 that the positioning performance of three smartphones is unsatisfactory in three locations. The reason is that pseudorange observations are affected by NLOS and multipath signals. In P1, the horizontal positioning error is about 14 m, and the vertical error is about 20 m. The positioning accuracy can be enhanced after using the proposed method to exclude NLOS. Specifically, there is a significant improvement on Huawei smartphones, while it is not obvious on MIA8. It could be caused by the fewer NLOS signals received by MIA8. In P2, there is a significant improvement in all smartphones after applying the method. Compared with P1, the improvement in P2 is greater; obviously, the reason is that the satellite geometry is better in P2, while satellites are blocked on one side in P1. In P3, the effectiveness of the method becomes limited, and positioning performance may even degrade. Furthermore, we can find that the positioning result cannot be improved even if all NLOSs are removed by a real skymask. The reason is that few LOS signals remain if we exclude all NLOS; a valid result cannot be obtained. Although excluding NLOS signals may reduce the number of available satellites and slightly degrade the positioning geometry (e.g., increase the DOP value), the large pseudorange errors introduced by NLOS propagation generally have a more severe impact on positioning accuracy. Therefore, removing severely obstructed NLOS signals usually leads to a more reliable positioning solution, even if the satellite geometry becomes slightly weaker. In this case, methods that mitigate NLOS effects would be suitable, such as the ray-tracing method or the shadow matching method [34,35].

Although the experiments in this study were conducted using static observations, the proposed skymask establishment and NLOS detection method is not restricted to static scenarios. The proposed method does not require any pre-existing 3D building model or external map information, because the skymask is inferred directly from GNSS observation time series and satellite ephemeris. The algorithm relies on GNSS observation time series and satellite ephemeris information, both of which are continuously available during dynamic positioning. In practical applications, the skymask can be gradually constructed using historical observation data and updated as new observations become available during receiver movement.

In addition, the computational steps involved in the proposed method mainly include satellite position calculation, observable-rate statistics, and smoothing-spline boundary fitting. These operations are computationally lightweight and can be efficiently implemented on modern smartphones. Nevertheless, dynamic environments may introduce additional challenges such as rapidly changing satellite visibility and receiver motion. Future work will further investigate the performance of the proposed method in dynamic smartphone positioning scenarios.

## 4. Discussion and Conclusions

Smartphone positioning is significantly affected by NLOS signals in the urban area, and a satisfactory positioning result cannot be obtained. Therefore, it is important to detect NLOS signals in this area. Several methods for NLOS detection have been proposed in the literature, including deep learning or 3D map-aided methods. However, there are some restrictions on NLOS detection on smartphones. For example, deep learning-based methods usually require labeled training datasets, which are difficult to obtain for daily smartphone positioning scenarios. For the 3D map-aided method, a 3D map is necessary, which cannot be obtained in some cities. In this paper, we propose an NLOS detection method that is free of extra information or datasets. We apply the time-series observations to establish the skymask in real-time and then classify the satellites by this skymask. The features of the method are as follows:

1. Based on precise ephemeris data, the positions of all satellites relative to the smartphone receiver are determined, and the observable rate of each satellite observation series is calculated for classification.
2. Using the categorized blocked and unblocked observation series, the building boundary is fitted with a smoothing spline, from which the skymask is extracted.
3. NLOS signals are detected using the extracted skymask. In addition, the same skymask can be used to estimate sky visibility and characterize the degree of signal obstruction.

The method is verified by the dataset collected in three locations by three smartphones, including a Huawei P40, a Huawei P30, and a Xiaomi Mi8. The effectiveness of the method is evaluated in four terms, including NLOS detection capacity, detection accuracy, sky visibility estimation, and positioning.

Based on the analysis, the number of NLOS signals increases sharply as the degree of obscuration increases, and the rate of NLOS in an epoch would reach 40%. In this case, the consistency checking method cannot work satisfactorily. After detecting and excluding NLOS signals using the proposed framework, the number of NLOS signals decreases significantly. Furthermore, we assess the detection accuracy of the method and compare it with the result of the C/N0 threshold detection method. The results of three smartphones indicate that the detection accuracy of the proposed method is stable in all locations. And the accuracy of the proposed method is 20% higher than that of detecting by the C/N0 threshold in the third location.

Sky visibility can be estimated by the established skymask. The results indicate that the effect of estimation on Huawei smartphones is better than Xiaomi Mi8, and the RMS of sky visibility estimation is 5–6% for Huawei phones, but more than 10% for Xiaomi Mi8 in certain cases. This could be caused by the difference in the capacity of signal reception. Finally, we analyzed the SPP performance on three smartphones, excluding NLOS, using the proposed method. The results indicate that the positioning performance can be enhanced when buildings are not dense. When the smartphone is blocked by buildings, it is preferable to mitigate NLOS signal effects using alternative methods compared to excluding NLOS signals directly.

Overall, the proposed method can be used to detect NLOS signals and estimate sky visibility using this approach. The method is useful for practical applications since it is free of extra equipment and information. As the NLOS cannot be detected by the method perfectly, it is suggested to use this method with consistency checking.

## Figures and Tables

**Table 1:** Information on smartphones used.

Station	Device	Antenna	GNSS Available
HP40	Huawei P40 (Huawei Technologies Co., Ltd., Shenzhen, China; HiSilicon-Kirin 990)	Embedded	GPS/BDS/ GLONASS/Galileo
HP30	Huawei P30 ((Huawei Technologies Co., Ltd., Shenzhen, China; HiSilicon-Kirin 980)	Embedded	GPS/BDS/ GLONASS/Galileo
MIA8	Xiaomi Mi8 (Xiaomi Corporation, Beijing, China; Broadcom BCM47755)	Embedded	GPS/BDS/ GLONASS/Galileo

**Table 2:** Results of single-point positioning after applying different methods.

Locations	Indexes	HP40			HP30			MIA8		
		E (m)	N (m)	U (m)	E (m)	N (m)	U (m)	E (m)	N (m)	U (m)
P1	Delete All	4.12	9.75	13.36	6.44	10.36	16.33	5.34	11.56	15.57
	CC	5.81	14.47	17.06	8.23	12.69	19.19	5.58	12.54	20.45
	Skymask	4.87	11.47	15.01	6.89	10.72	17.44	5.50	12.37	20.18
	Imp	16.2%	20.7%	12.0%	16.3%	15.5%	9.1%	1.4%	1.4%	1.3%
P2	Delete All	6.62	10.17	29.3	9.31	10.57	33.39	6.08	8.98	19.47
	CC	8.07	10.62	61.38	13.66	20.35	66.39	7.14	10.43	47.08
	Skymask	5.35	9.43	18.91	10.62	10.88	36.05	6.14	6.91	21.36
	Imp	33.7%	11.2%	69.2%	22.3%	46.5%	45.7%	14.0%	33.7%	54.6%
P3	Delete All	11.83	24.05	33.57	12.09	16.56	29.66	14.50	24.90	30.23
	CC	11.89	24.20	33.61	12.14	17.35	33.16	14.20	25.08	29.85
	Skymask	11.87	24.70	33.01	10.86	16.40	27.08	14.92	26.14	29.09
	Imp	0.2%	−2.1%	1.8%	10.5%	5.5%	18.3%	−5.1%	−4.2%	2.5%

## Data Availability

The data presented in this study are available on request from the corresponding author.
